# Dr. Carter N. Berkeley, of Philadelphia—Dr. Yellowly, of England

**Published:** 1842-04-30

**Authors:** 


*Obituary.*—We record with great regret the death of our friend and correspondent, Dr. Carter N. Berkeley, of this city. Dr. Berkeley was a native of Hanover county, Va., but had been established in Philadelphia for some years, with the best prospects of professional success. His career was arrested about a year since by the first attack of the disease to which he finally fell a victim on the 16th of the present month. Dr. Berkeley was universally esteemed and beloved. His abilities were excellent; his devotion to his profession entire; his character most honourable and upright; his disposition and manners uncommonly attractive. He is generally and sincerely lamented.

Among the deaths of physicians of note abroad, we notice that of Dr. Yellowly, formerly physician to the London Hospital. He was one of the founders of the Medico-Chirurgical Society, with Dr. Marcet, Sir A. Cooper, and a few others; also, one of the promoters and principal supporters of the British Association, and a frequent contributor to the Medico-Chirurgical and Philosophical Transactions. Dr. Y. died on the 31st of January last, in his 65th year.

